# Electrochemical Biosensor Using Nitrogen-Doped Graphene/Au Nanoparticles/DNAzyme for Ca^2+^ Determination

**DOI:** 10.3390/bios12050331

**Published:** 2022-05-12

**Authors:** Zhixue Yu, Hui Wang, Yiguang Zhao, Fan Zhang, Xiangfang Tang, Benhai Xiong

**Affiliations:** 1 State Key Laboratory of Animal Nutrition, Institute of Animal Sciences, Chinese Academy of Agricultural Sciences, Beijing 100193, China; 82101205310@caas.cn (Z.Y.); wanghui10@caas.cn (H.W.); zhaoyiguang@caas.cn (Y.Z.); 82101199124@caas.cn (F.Z.); tangxiangfang@caas.cn (X.T.); 2 College of Animal Science and Technology, China Agricultural University, Beijing 100193, China

**Keywords:** electrochemical biosensor, nitrogen-doped graphene, gold nanoparticles, DNAzyme, dairy cow, hypocalcemia

## Abstract

An electrochemical biosensor for detecting Ca^2+^ concentration was proposed using glass carbon electrodes (GCEs) modified with nitrogen-doped graphene (NGR), gold nanoparticles (AuNPs) and DNAzyme. The resistance signal was amplified through two methods: electrochemical reduction of AuNPs on the NGR surface to increase the specific surface area of the electrode and strengthen the adsorption of DNAzyme; and increasement of the DNAzyme base sequence. The process of electrode modification was characterized by scanning electron microscopy, Raman spectroscopy, cyclic voltammetry (CV), and electrochemical impedance spectroscopy (EIS). Experimental parameters’ influence, such as the deposition time of gold nanoparticles and the detection time, were assessed by electrochemical methods. The linear ranges of the electrochemical biosensor were in the range from 5 × 10^−6^ to 5 × 10^−5^ and 5 × 10^−5^ to 4 × 10^−4^ M, with a detection limit of 3.8 × 10^−6^ M. The concentration of Ca^2+^ in the serum of dairy cows was determined by the biosensor with satisfactory results, which could be potentially used to diagnose subclinical hypocalcemia.

## 1. Introduction

DNAzyme is a single-stranded DNA sequence with the advantages of simple synthesis, high stability and fast reaction speed. At present, DNAzyme has broad application prospects in the highly selective recognition of elements by metal ions [1]. DNAzymes are selectively isolated in vitro using Ca^2+^ [2], Pb^2+^ [3], Cu^2+^ [4], Zn^2+^ [5], Mg^2+^ [6], UO_2_^2+^ [7], and Hg^2+^ [8] as cofactors and have high specificity for metal ions [9]. DNAzyme consists of the catalytic part and binding arm [10]. There is an adenosine ribonucleotide (rA) cleavage site in the substrate chain [11]. The reason why DNAzyme is selected to measure calcium ions is that when the binding arm and the substrate chain are hybridized into a double chain structure through base complementary pairing, calcium ions, as a cofactor, will specifically combine with the catalytic center to initiate the catalytic activity of the enzyme, prompting the enzyme to recognize and crack the phosphate diester bond in the substrate chain [2]. Heaton et al. [12] quantified Ca^2+^ by measuring structural changes in the binding of Ca^2+^ and DNAzymes. Zhou et al. [13] reported a DNAzyme named EtNa, which could be specifically activated by Ca^2+^ with a detection limit of 17 μM Ca^2+^, showing good selectivity. They also selected DNAzyme in serum and obtained 17EV1, which was more active with Ca^2+^ [14].

Graphene, a two-dimensional material, has broad application prospects in the fields of bioelectronics and biosensing. Graphene is widely used in electronic devices and biosensors because of its unique physical and chemical properties, such as high specific surface area, excellent electrical conductivity, easy production, and functionalization [15,16]. In order to improve the characteristics of graphene in bioelectrochemical applications, chemical doping is the primary potential strategy carried out to increase the density of free carriers and improve conductivity [17]. Studies demonstrate that nitrogen is a good element for the chemical doping of carbon materials because it has a comparable atomic size and contains five valence electrons, forming strong valence bonds with carbon atoms [18]. Compared with original graphene, nitrogen-doped graphene has larger specific surface area, higher volume ratio of surface-active groups, and stronger electrocatalysis. It has been widely used in electrochemical catalysis and biosensors [19,20,21,22].

To improve the performance of biosensors, it is necessary to improve the efficiency of DNA hybridization and increase the fixed amount of biomaterials. Nanomaterials play an important role in the adsorption and fixation of biomolecules [23,24]. Among metal nanoparticles, gold nanoparticles (AuNPs) are considered to be candidate materials for electrochemical sensors because of their excellent electrocatalytic activity. The presence of nitrogen can promote the high dispersion of AuNPs and induce excellent conductivity [25]. The combination of AuNPs and NGR increases the specific surface area of the electrode and improves the catalytic performance of the electrons. High specific surface area allows for more fixation of probe DNA [26]. In addition, organic thiolated compounds and thiolated oligonucleotides can react on the gold surface to form S-Au covalent bonds, which improves the sensitivity and selectivity of biosensors [27,28,29].

Hypocalcemia is a common nutritional and metabolic disease in dairy cows before and after parturition in intensive dairy farms in China. After calving, a large amount of blood calcium is used to synthesize milk, which can cause hypocalcemia in dairy cows. Clinical hypocalcemia is often accompanied by muscle weakness, anorexia, quadriplegia and other apparent symptoms, and even coma or death. Subclinical hypocalcemia without symptoms can induce postpartum paralysis, ketosis, fetal failure, gastric displacement, uterine prolapse, and other diseases, and can reduce the milk yield and service life and increase the mortality of dairy cows [30,31,32]. According to statistics, the average incidence of clinical hypocalcemia in dairy cows is 7.5%, and the incidence of subclinical hypocalcemia is about 30% [33]. Due to a lack of practical, rapid, and inexpensive on-site detection techniques, subclinical hypocalcemia is easily ignored by breeding farms. Therefore, the fast and economical detection of Ca^2+^ concentration in the serum of dairy cows would be highly useful in diagnosing subclinical hypocalcemia.

The traditional Ca^2+^ analysis method based on spectral analysis has high sensitivity and precision. However, this method can only be carried out in specific laboratories operated by trained technicians, and the sample pretreatment is time-consuming and expensive, so it is not suitable for rapid on-site monitoring [34]. Therefore, these highly instrument-dependent detection methods cannot fully meet the needs of rapid real-time monitoring. Compared with instrumental detection methods, electrochemical biosensors are characterized by simplicity, rapidity, low cost, and high sensitivity [35,36,37]. Although the ion-selective electrode (ISE) has good selectivity and a short detection time, the measurement of actual samples is mainly limited by the unstable signal generated by the complex matrix. When using the ISE to measure Ca^2+^ concentration [38], Cu^2+^ in natural water [39], Hg^2+^ in seawater [40], and Pb^2+^ in drinking water [41], as with all classic ISEs with internal solutions, this electrode is limited to vertical measurements, difficult to miniaturize, and leaks ions from internal solutions [42]. In addition, the mechanical strength and durability of the ion-sensitive membrane are insufficient, and if the ion-sensitive membrane is broken, the internal solution can also leak into the analyte [43]. Although these disadvantages can be overcome by using solid-state electrodes, solid-state electrodes are often affected by pH and the formation of an aqueous layer, resulting in signal drift and distortion, which can reduce the measurement accuracy [44,45]. When commercial ISEs are used to measure real samples, the electrical signals fluctuate greatly and are unstable, which indicates that it is difficult to use classical commercial ISEs to measure real complex samples. An AuNPs/NGR nanohybrid based on DNAzyme is a new electrocatalyst for serum Ca^2+^ detection with high sensitivity, high selectivity, and good reproducibility. In this work, we developed an electrochemical biosensor fabricated using glass carbon electrodes modified with nitrogen-doped graphene, gold nanoparticles, and DNAzyme, which was then applied to diagnose subclinical hypocalcemia in dairy cows. 

## 2. Materials and Methods

### 2.1. Materials and Reagents

Nitrogen-doped graphene (NGR) was bought from Shanghai Aladdin Biochemical Technology Co., Ltd. (Shanghai, China). 6-Mercapto-1-hexanol (MCH), N, N-Dimethylformamide (DMF), Sodium acetate, NaCl, KCl, MgCl_2_, CaCl_2_, CuCl_2_, FeCl_2_, FeCl_3_, and ZnCl_2_ were provided by Sigma Aldrich (Beijing, China). HAuCl_4_, Na_2_SO_4_, Tris (2-carboxyethyl) phosphine (TCEP), and ethanol were purchased from Macklin Inc. (Shanghai, China). Tris (hydroxymethyl) ammonia methane (Tris) was provided by Titan Scientific Co., Ltd. (Shanghai, China). Both hydrochloric acid (HCl) and acetic acid (CH_3_COOH) came from Beijing Chemical Co., Ltd. (Beijing, China). The Ca^2+^ standard solution was provided by the National Research Center for Reference Materials of China (Beijing, China) and diluted to the required Ca^2+^ concentration. All chemical reagents in this study were analytically pure without further purification. Millipore Milli-Q Systems produced ultra-pure water. Tris-HCl buffer was used. Oligonucleotide sequences were synthesized by Sangon Biotechnology Co., Ltd. (Shanghai, China). Table 1 shows the oligonucleotide sequences. 

The substrate chain (Sub) required sulfhydryl reduction: 4 mg TCEP was dissolved in 100 µL Tris-HCl solution and then mixed thoroughly with 100 µL Sub solution. The mixture was left for 1 h until 200 µL acetic acid solution of 6% and 50 µL 3 M sodium acetate were added. It was then centrifuged at 5000 rpm at room temperature for 5 min to remove the supernatant, and the remains were dispersed in sterile water to a concentration of 50 µM. Sub combined with DNAzyme to form the Sub-DNAzyme duplex.

### 2.2. Apparatus and Measurements

SEM images were obtained by field emission scanning electron microscope (FE-SEM SU8040) at 10 kV acceleration voltage. Raman spectra were measured using Renishaw inVia using an imaging microscope. Electrical signals were recorded using a CHI 760E Electrochemical Station (Shanghai Chenhua Instrument, Shanghai, China) with three electrodes. In this recording, the modified electrode (GCE/NGR/AuNPs/Sub-DNAzyme duplex), platinum electrode, and Ag/AgCl electrode were used as the working electrode, reference electrode, and counter electrode, respectively. The centrifuge (SN-LSC-40) was provided by Shanghai Shangyi Instrument Equipment Co., Ltd. (Shanghai, China).

### 2.3. Fabrication 

**Figure 1:**
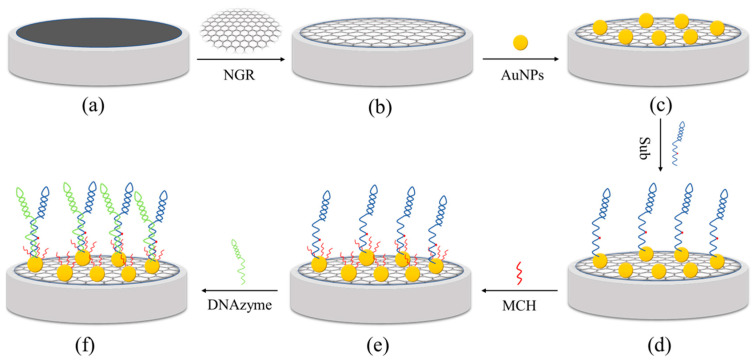
The process of GCE (**a**) functionalized with NGR (**b**), AuNPs (**c**), Sub (**d**), MCH (**e**), and DNAzyme (**f**).

Figure 1 describes the modification process of the GCE/NGR/AuNPs/Sub-DNAzyme duplex. Briefly, the bare GCE was polished with 0.3 µm and 0.05 µm Al_2_O_3_ powder, followed by multiple ultrasonic treatments in deionized water. The surface of the GCE covered with 6 μL 0.01 M NGR solution was dried at 60 °C for 30 min, and was then rinsed with sufficient water to remove the residual NGR. AuNPs were electrodeposited by cyclic voltammetry. The GCE/NGR/AuNPs was formed by placing GCE/NGR in 5 mL 0.3 mM HAuCl_4_ and 0.01 M Na_2_SO_4_ solution. Then, 10 cycles were scanned at a rate of 100 mV/s in the potential range of −0.2 to 1.2 V. The GCE/NGR/AuNPs was covered with 5 μL 50 μM Sub solution at high humidity overnight at 4 °C. During this time, Sub could be covalently connected to the AuNPs’ surface by forming S-Au bonds. Drops of 0.1 mM MCH were applied to the surface of the modified electrode to prevent adsorption of unbound Sub on the Au surface. Finally, Sub on Au surface hybridized with DNAzyme to form the GCE/NGR/AuNPs/Sub-DNAzyme duplex for 4 h. DNAzyme could bind to Ca^2+^ and then cleave complementary substrates (Sub) at the rA site. Because the conductivity of the Sub-DNAzyme duplex was relatively poor, the structure could affect the resistance of the GCE/NGR/AuNPs, thus facilitating highly selective Ca^2+^ detection.

### 2.4. Method of Measurement

To measure Ca^2+^ concentration, *R_ct_* values need to be calculated by electrochemical biosensor before and after measuring the real samples. The Ca^2+^ was covered on the biosensor for 15 min and then rinsed with an adequate quantity of water to remove the excess. The sensor was then immersed in 5 mM [Fe(CN)_6_]^4−/3−^ and 0.1 M KCl for EIS.

The calculation formula of relative resistance is as follows:

(1) $$\mathrm{Relative}\;\mathrm{resistance}=\frac{\;R_{ct0}-R_{ct}}{\;R_{ct0}}\times 100\%\;$$

*R_ct_*_0_ is to measure the initial resistance value, *R_ct_* is to measure the resistance value of the actual sample.

### 2.5. Measurement of Real Samples

Blood samples from dairy cows were collected at the China-Israel demonstration dairy farm in southeast Beijing. A 10 mL centrifuge tube containing heparin lithium was used to collect blood samples from the tail vein of dairy cows. The blood samples were centrifuged at 5000 rpm at room temperature for 5 min, and the supernatant was then separated, which was diluted four times using 0.1 M Tris-HCl buffer solution with pH 7.4.

## 3. Results

### 3.1. Characterization

**Figure 2:**
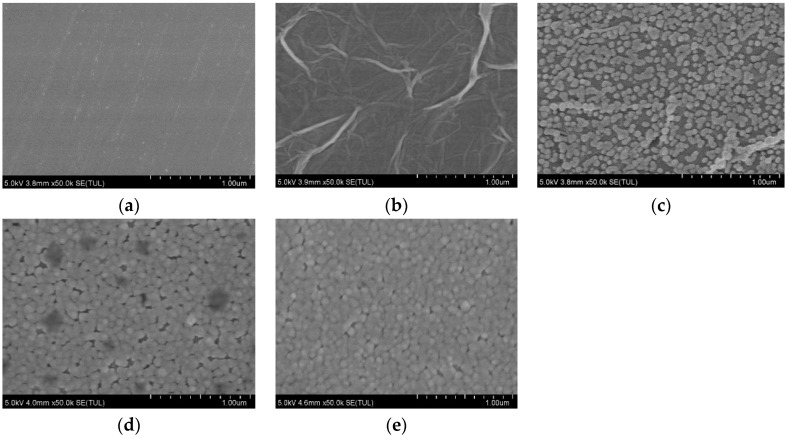
SEM images of bare GCE (**a**), GCE/NGR (**b**), GCE/NGR/AuNPs (**c**), GCE/NGR/AuNPs/Sub (**d**), and GCE/NGR/AuNPs/Sub-DNAzyme duplex (**e**).

SEM was used to characterize the structure and micromorphology of the GCE before and after being modified with NGR, AuNPs, Sub, and DNAzyme, shown in Figure 2. As shown in Figure 2a, bare GCEs polished using 0.3 µm and 0.05 µm Al_2_O_3_ powder had a smooth surface. The SEM image of GCE/NGR in Figure 2b was covered with multilayer film and accompanied by a folded structure, suggesting that NGR had been deposited on the GCE. The NGR was deposited on the electrode surface, which effectively increased the specific surface area of the sensing interface and provided more active sites for AuNPs deposition [46]. In Figure 2c, there were dense nanoparticles present on the surface of NGR after electroplating chloroauric acid solution, which can improve the specific surface area [47]. When the GCE/NGR/AuNPs was functionalized with Sub and DNAzyme, the structure was as shown in Figure 2d,e. Because Sub and DNAzyme were non-conductive materials with size, when they were modified on the surface of gold nanoparticles, the size increased. Due to poor conductivity, SEM imaging was affected under the same conditions, so the image looked blurred, which was consistent with previous reports [48].

**Figure 3:**
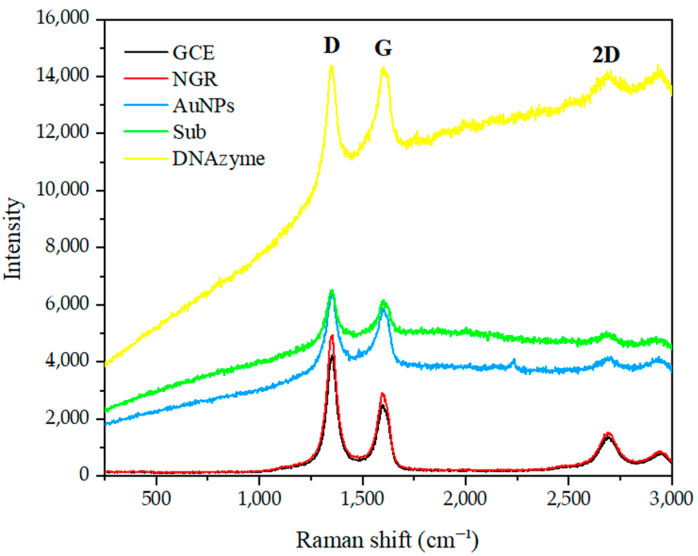
Raman spectrum of GCE modified with NGR, AuNPs, Sub and DNAzyme.

As shown in Figure 3, Raman spectroscopy was used to study the changes of various GCE-modified materials under 532 nm laser excitation. The Raman spectra of GO contained three peaks located at 1352 cm^−1^, 1592 cm^−1^, and 2690 cm^−1^, corresponding to the D, G, and 2D bands, respectively. After the NGR was immobilized on the GCE, the high-intensity D band at 1352 cm^−1^ indicated many defects in the NGR layer. These defects could be attributed to damage of *sp*^2^ C and nitrogen doping [49]. A sharp G-band at 1592 cm^−^^1^ indicated that the nitrogen-doped graphene maintained its two-dimensional structure. At 2690 cm^−1^, a wideband corresponding to the design of nitro fossilized graphene was observed, which was consistent with the report of Sheng et al. [50], further confirming the existence of graphene structure in the final product. When AuNPs, Sub, and DNAzyme were used to treat GCE/NGR, the intensity ratio of I_D_/I_G_ decreased (1.69, 1.58, 1.38), respectively. It is possible that the modified molecules interacted with each other, and that the molecules’ influence on light weakened the electric field and reduced the signal.

### 3.2. CV and EIS Characterization

**Figure 4:**
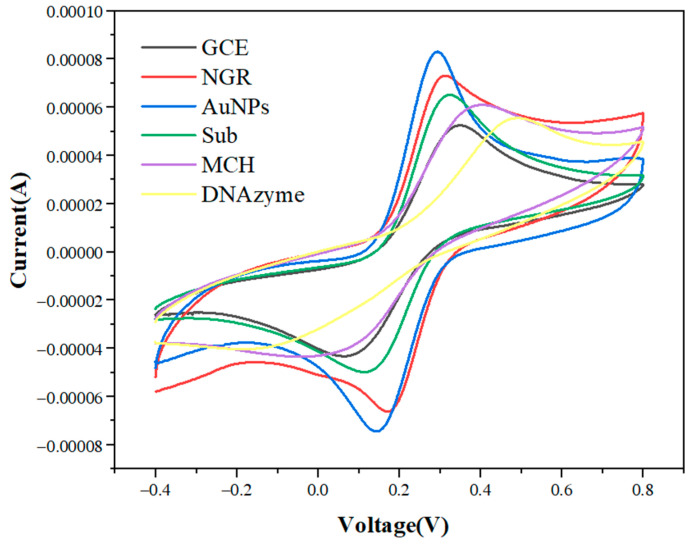
Cyclic voltammetry curves of GCE modified with NGR, AuNPs, Sub, MCH, and DNAzyme in 5.0 mmol/L [Fe(CN)_6_]^4^^−/3^^−^ and 0.1 mol/L KCl at 50 mV/s scanning rate.

Figure 4 shows the cyclic voltammetry curves of GCE functionalized successively by NGR, AuNPs, Sub, MCH, and DNAzyme in 5 mmol/L [Fe(CN)_6_]^4−/3−^ and 0.1 mol/L KCl mixed solution. When NGR was fixed on the GCE surface, the peak current increased from 52 μA to 73 μA. The peak current of AuNPs-modified GCE/NGR increased to 83 μA. These phenomena indicated that conductive nanomaterials could improve the specific surface area and the electron transfer rate between the electrode and the solution. When Sub with negative charge was assembled on the AuNPs surface, the peak current decreased because the negative direction of current and the negative charge of Sub sequence induced electrostatic repulsion on the electrode surface in [Fe(CN)_6_]^4−/3−^, thus preventing the electron transfer rate [51]. When MCH blocked AuNPs and NGR non-specific sites, the peak current decreased. Finally, the peak current was significantly reduced when the electrode was placed in 50 mM DNAzyme. This was due to the large negative charge generated by DNAzyme, which repelled the transfer rate of [Fe(CN)_6_]^4−/3−^ between electrode and electrolyte [52]. In addition, immobilization of Sub and DNAzyme resulted in increased electrode surface density and prevented electron transfer between the electrode surface and electrically-active species.

**Figure 5:**
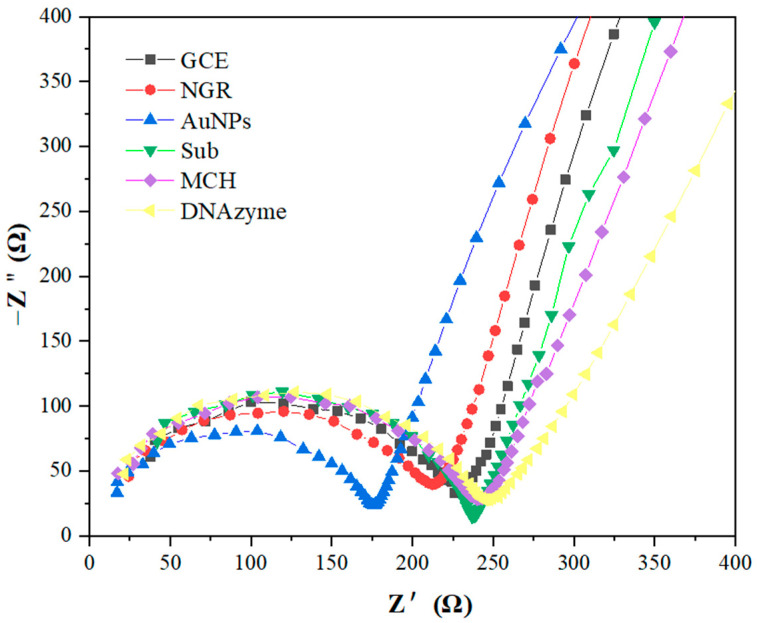
In 5 mmol/L [Fe(CN)6]^4−/3−^ and 0.1 mol/L KCl solution, GCE modified with NGR, AuNPs, Sub, MCH, and DNAzyme obtained EIS in the frequency range from 0.1 to 10^5^ Hz (potential = 0.2 V).

Electrochemical impedance spectroscopy (EIS) was used to monitor the resistance characteristics of GCE-modified NGR, AuNPs, Sub, MCH, and DNAzyme in 5 mM [Fe(CN)_6_]^4−/3−^ and 0.1 M KCl at room temperature. The typical Nyquist resistance spectrum includes a semicircle at high frequencies corresponding to electron transfer confinement processes. The linear part at low frequencies corresponds to diffusion-constrained processes. As shown in Figure 5, *R_ct_* of resistance spectrum on bare GCE was about 230 ± 11 Ω. When NGR was deposited on the surface of the GCE, the semicircles in the resistance spectrum decreased due to the good electrical conductivity of deposited NGR. After AuNPs were assembled on the GCE/NGR surface, the semicircles in the resistance spectrum decreased significantly, indicating that the charge transfer process was fast. When Sub functionalized on the Au surface via the S-Au bond, the resistance value increased, and the *R_ct_* reached 238 ± 9 Ω. The negatively charged probe sequence fixed on the electrode surface generated repulsion with the negatively charged redox probe [Fe(CN)_6_]^4−/3−^ [47]. The subsequent surface blocking of MCH also significantly increased *R_ct_* to 244 ± 13 Ω, indicating that MCH occupied the remaining bare position of unspecific adsorption [53]. After DNAzyme hybridization, the *R_ct_* value was 250 ± 13 Ω. The resistance of the GCE/NGR/AuNPs/Sub-DNAzyme duplex increased slightly, but the relative resistance decreased noticeably when exposed to different Ca^2+^ concentrations in Section 3.5, indicating DNAzyme was hybridized with Sub successfully. EIS results were consistent with CV, so it could be concluded that the biosensor was successfully prepared.

### 3.3. Optimization

To improve the sensitivity of the electrochemical sensors, some detection parameters were optimized at different Ca^2+^ concentrations.

**Figure 6:**
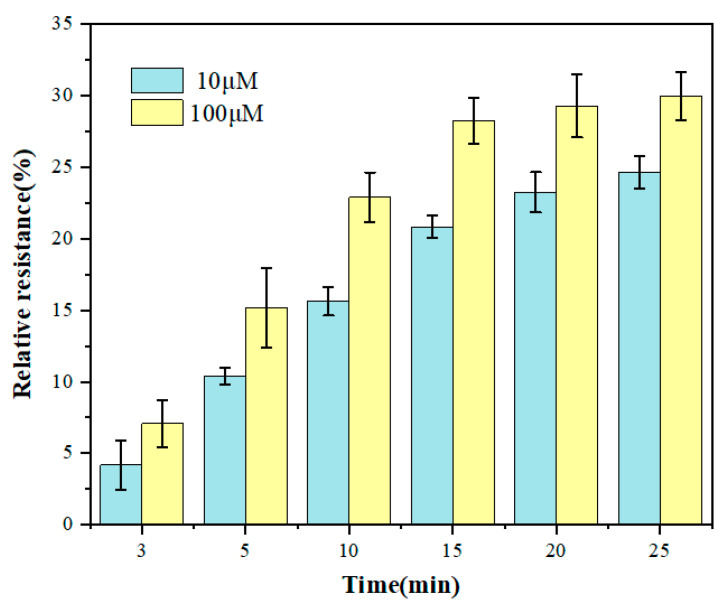
The relative resistance of the GCE/NGR/AuNPs/Sub-DNAzyme duplex after incubation in different Ca^2+^ concentrations at different times (The error bars were determined in three duplicates).

Incubation time had a significant influence on the sensitivity of the electrochemical biosensor. Figure 6 shows the change of relative resistance of the GCE/NGR/AuNPs/Sub-DNAzyme duplex at different Ca^2+^ concentrations with incubation time. The relative resistance of DNAzyme increased significantly with the incubation time ranging from 0 min to 15 min, which was due to the effect of Ca^2+^ on the continuous cleavage of DNAzyme. However, when the incubation time exceeded 15 min, the response signal was relatively stable, especially at the concentration of 100 μM Ca^2+^, indicating that the adsorption on the surface of GCE had reached saturation. To balance the sensitivity of biosensor and incubation time, 15 min was selected as the best incubation time.

**Figure 7:**
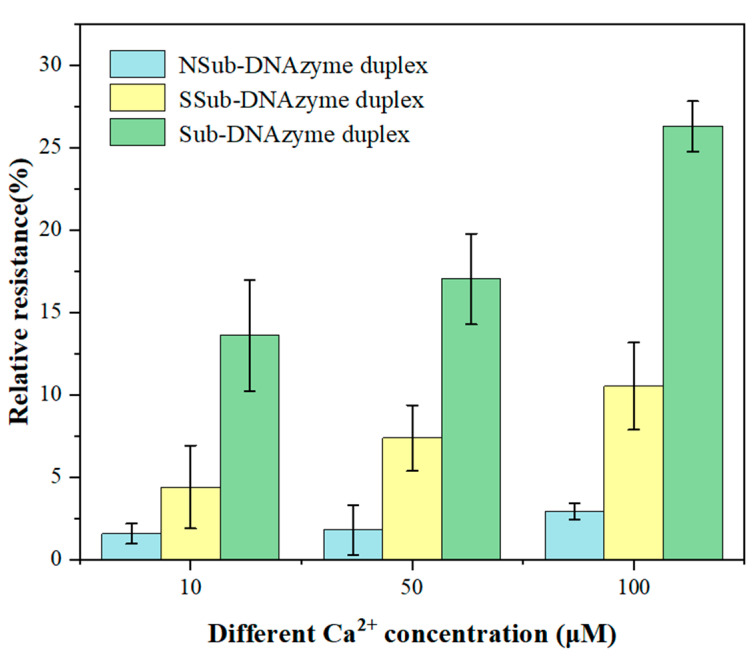
The relative resistance of Sub-DNAzyme duplex structure and length to different Ca^2+^ concentration (The error bars were determined in three duplicates).

The structure and length of Sub and DNAzyme that combine to form the Sub-DNAzyme duplex are two key factors affecting the sensitivity of the GCE/NGR/AuNPs/Sub-DNAzyme duplex. Figure 7 shows the relative resistance of different Sub-DNAzyme duplexes (NSub-DNAzyme duplex, SSub-DNAzyme duplex, and Sub-DNAzyme duplex) for 10, 50, and 100 μM Ca^2+^. When combined with Ca^2+^, DNAzyme catalyzed the cleavage of Sub on the electrode surface at rA, which reduced the substrate chain density on the electrode surface and reduced the charge transfer resistance. The relative resistance of the NSub-DNAzyme duplex without rA was almost zero, suggesting that DNAzyme bound with Ca^2+^ did cleave Sub at rA. The relative resistance of the SSub-DNAzyme duplex and Sub-DNAzyme duplex-modified electrodes increased with the increase of Ca^2+^ concentration. However, the relative resistance of the SSub-DNAzyme duplex was not as high as that of the Sub-DNAzyme duplex. This suggested that the length of DNAzyme may affect the sensitivity of the GCE/NGR/AuNPs/Sub-DNAzyme duplex. Therefore, the combination of DNAzyme and Sub were used to measure Ca^2+^ concentration in this experiment.

**Figure 8:**
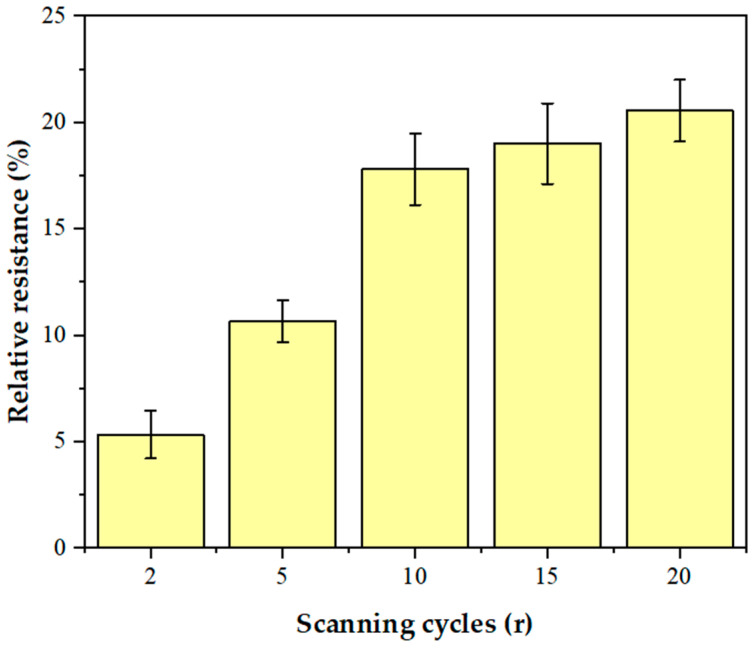
The relative resistance of GCE/NGR/AuNPs/Sub-DNAzyme duplex electrodeposition of AuNPs with different scanning cycles (2 r, 5 r, 10 r, 15 r, and 20 r) in 100 μM Ca^2+^ solution (The error bars were determined in three duplicates).

The number of AuNPs has a significant effect on the electrochemical performance of the sensor. In order to optimize the electrodeposition conditions of AuNPs, different amounts of AuNPs were deposited on the GCE/NGR surface by controlling the number of scanning cycles (2 r, 5 r, 10 r, 15 r, and 20 r). Figure S1 shows the deposition process of AuNPs. Figure 8 shows the change of relative resistance of the GCE/NGR/AuNPs/Sub-DNAzyme duplex at 100 μM Ca^2+^ concentration with different scanning cycles of AuNPs. The sensitivity of the sensor increased significantly as the scanning cycles increased from 2 r to 10 r, and then remained stable. As the number of scanning cycles increased, AuNPs became larger and more uniform, which was not conducive to electrochemical catalysis [54]. The GCE/NGR/AuNPs/Sub-DNAzyme duplex sensor showed the highest sensitivity at the scanning cycles of 10 r. Therefore, 10 r was chosen as the optimal scanning cycle. 

Figure S2 shows the comparison of relative resistance measurement between AuNPs modified on NGR surface and naked GCE surface under different Ca^2+^ concentrations. As shown in the comparison figure, the relative resistance of the electrode modified with NGR was higher than that not modified. The reason is that the NGR increased the specific surface area of the electrode, making more AuNPs bond to the electrode surface and increasing the conductivity of the electrode. Therefore, the combination of NGR and AuNPs were selected to produce the electrode.

### 3.4. Interference

**Figure 9:**
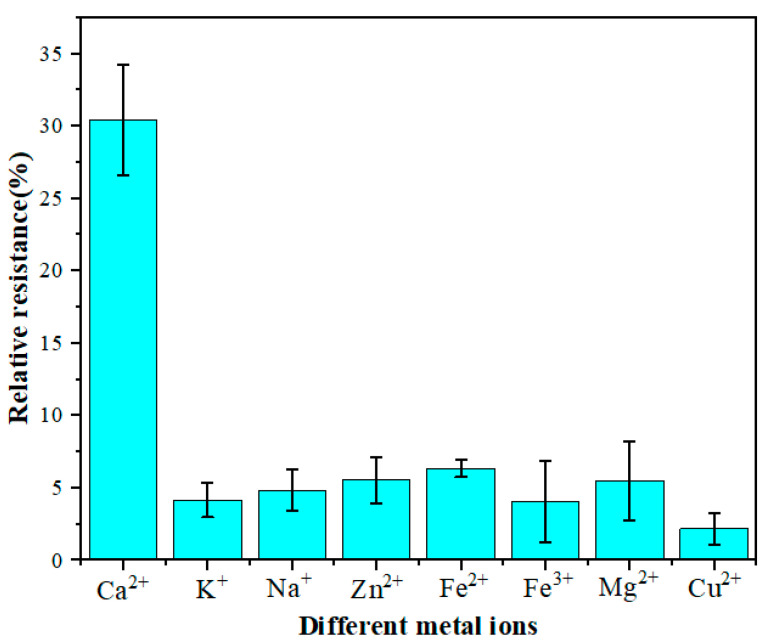
The relative resistance of GCE/NGR/AuNPs/Sub-DNAzyme duplex incubated in different 0.4 mM metal ion solutions (K^+^, Na^+^, Zn^2+^, Fe^2+^, Fe^3+^, Mg^2+^, and Cu^2+^) (The error bars were determined in three duplicates).

Anti-interference is an essential factor affecting the performance of electrochemical biosensors. There are many metal ions in the serum of dairy cows, which may interfere with the relative resistance of the GCE/NGR/AuNPs/Sub-DNAzyme duplex. Therefore, seven metal ions with high concentrations were selected, including K^+^, Na^+^, Zn^2+^, Fe^2+^, Fe^3+^, Mg^2+^, and Cu^2+^. The GCE/NGR/AuNPs/Sub-DNAzyme duplex was then used to detect 0.4 mM of these metal ions in the 0.1 M Tris-HCl at pH 7.4. The results in Figure 9 show that the relative resistance of the GCE/NGR/AuNPs/Sub-DNAzyme duplex to 0.4 mM Ca^2+^ was about 30%, but the relative resistance of other metal ions was much less than Ca^2+^, indicating that these metal ions had no interference effect on the GCE/NGR/AuNPs/Sub-DNAzyme duplex.

### 3.5. Ca^2+^ Sensing

**Figure 10:**
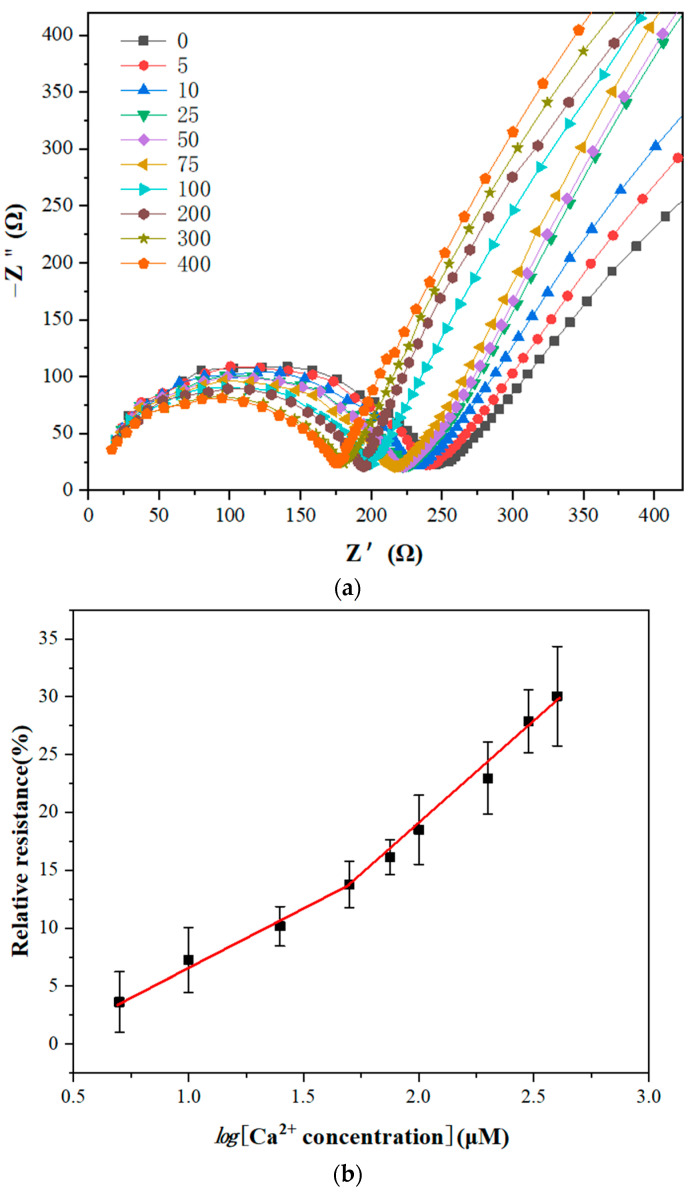
(**a**) Electrochemical impedance spectroscopy of GCE/NGR/AuNPs/Sub-DNAzyme duplex at different Ca^2+^ concentrations from 0 μM to 400 μM; (**b**) Linear relationship between relative resistance and logarithms of Ca^2+^ concentration (The error bars were determined in three duplicates).

To evaluate the analytical performance of the GCE/NGR/AuNPs/Sub-DNAzyme duplex, the EIS was recorded by analyzing a series of Ca^2+^ concentrations under optimal conditions, and three measurements were repeated for each concentration. The semicircle diameter decreased with the increase of Ca^2+^ concentration in Figure 10a. The linear relationship between Ca^2+^ concentration and relative resistance ranged from 0 μM to 400 μM. The calculated relative resistance is shown in Figure 10b. When Ca^2+^ concentration was less than 50 μM, the relative resistance increased slowly, indicating that the cleavage efficiency of the Sub-DNAzyme duplex was low at low Ca^2+^ concentration. The logarithmic relationship between the relative resistance and Ca^2+^ concentration was linear in the range of 5 to 50 μM. The linear equation was relative resistance = −2.9637 + 9.7288 log[Ca^2+^](µM) and the linear regression coefficient was 0.9901. In the range of 50 to 400 μM, the relative resistance increased rapidly with the increase of Ca^2+^ concentration. The linear equation was relative resistance = −17.862 + 18.252 log[Ca^2+^](µM), and the linear regression coefficient was 0.9893.

Table 2 lists the detecting parameters of Ca^2+^ by different biosensors. Most DNAzyme-modified sensors have a similar linear range. Although the linear range of the electrochemical device (GCE/NGR/AuNPs/Sub-DNAzyme duplex) developed in this paper was narrower than that of some sensors and the detection limit was not low enough, for cows, the serum Ca^2+^ concentration was about 1 to 1.25 mM and 250 to 300 μM after diluting four times. Therefore, the electrochemical device didn’t need a wide linear range and a very low detection limit to detect serum Ca^2+^ in dairy cows. In addition, serum Ca^2+^ could be detected without complex pretreatment, and the detection time was 15 min, which was shorter than UV-vis to detect Ca^2+^, meeting the needs of practical application.

### 3.6. Determination of Ca^2+^ in Real Samples

An electrochemical instrument was used to test the serum samples of dairy cows with different concentrations, following the preprocessing procedure of the blood sample described in Section 2.5. The method developed in the current study and an atomic absorption spectrometry (AAS) were used to detect the same extracted samples. The results are shown in Table 3. Although the detection accuracy of our method was slightly lower than that of the AAS, the recoveries ranged from 93.48% to 104.90%, which indicated its suitability in determining Ca^2+^ concentration in serum samples. A *t*-test was performed on the results of the two methods, and *p* = 0.78 > 0.05. There was no significant difference between the results obtained by the two methods, indicating that the biosensor was accurate and repeatable. It could be more practically used to determine Ca^2+^ concentration in the serum of dairy cows at farms.

## 4. Conclusions

This study developed a novel DNA electrochemical biosensor based on the GCE/NGR/AuNPs/Sub-DNAzyme duplex. NGR/AuNPs-modified GCE not only increased the specific surface area of the electrode, but also accelerated the electron transfer and enhanced the signal detection. Under optimal conditions, the linear ranges of the electrochemical biosensor were 5 × 10^−6^ to 5 × 10^−5^ and 5 × 10^−5^ to 4 × 10^−4^ M, showing high selectivity and sensitivity. In addition, the electrochemical biosensor had good stability, a short detection time, and didn’t require an extremely low detection limit. Therefore, it provided a practical option for the detection of Ca^2+^ in cow serum, which may contribute to the diagnosis of subclinical hypocalcemia at dairy farms.

## Figures and Tables

**Table 1:** The modified DNA sequences.

DNA	Sequences and Modifications (Start with 5 Terminal ′)
Sub	HS-SH-(CH_2_)_6_-GCTGTAGAAGG/rA/TATCACTGTGCACTAAGCGGTA GAACTCACTATGTAGTGAGTTCTACCGCT
SSub	HS-SH-(CH_2_)_6_-GCTGTAGAAGG/rA/TATCACTGTGCACTAA
NSub	HS-SH-(CH_2_)_6_-GCTGTAGAAGGATATCACTGTGCACTAAGCGGTA GAACTCACTATGTAGTGAGTTCTACCGCT
SDNAzyme	TAGTGCACAGTGATTGTTGGAATCGCTCATGCGACACTC TTTTCTACAGC
DNAzyme	TCGCCATCTTGAGTGTTACAGCACTCAAGATGGCGATAGTGCACAGTGATTGTTGGAATCGCTCATGCGACACTCTTTTCTACAGC

**Table 2:** Comparison of GCE/NGR/AuNPs/Sub-DNAzyme duplex performance with other reported sensors for Ca^2+^ determination.

Sensor	Method	Linear Range(M)	Hybridization Time (min)	Detection Limit (M)	Ref.
DNAzyme/AuNPs	SWV	10^−^^7^~10^−^^3^	90	10^−^^7^	[55]
GCE/SWNT/DNAzyme	EIS	5 × 10^−^^6^~2.5 × 10^−^^2^	7	4.2 × 10^−6^	[56]
DNAzyme/SWNT/FET	EIS	7.5 × 10^−^^6^~10^−^^3^	9	5.48 × 10^−^^6^	[57]
CD-EGTA *	Fluorescence	1.5 × 10^−5^~3 × 10^−4^	-	3.8 × 10^−7^	[58]
EICON *	UV-vis	1 × 10^−4^~5 × 10^−4^ 1 × 10^−3^~5 × 10^−3^	30	6 × 10^−5^	[59]
CSS-PAH	UV-vis	5 × 10^−4^~1	35	5 × 10^−4^	[60]
GCE/NGR/AuNPs/Sub-DNAzyme duplex	EIS	5 × 10^−6^~5 × 10^−5^ 5 × 10^−5^~4 × 10^−^^4^	15	3.8 × 10^−6^	This work

* CD-EGTA: carbon dot-ethylenebis (oxyethylenenitrilo) tetraacetic acid; EICON: entrapping ionophore based calcium-selective organosilica nanoparticles.

**Table 3:** Comparison of electrochemical device and AAS in determining Ca^2+^ concentrations in dairy cow serum samples.

Sample	Electrochemical Device (μM)	AAS (μM)	Recovery (%)
1	933 ± 42	983 ± 12	94.90 ± 4.2
2	1070 ± 51	1021 ± 15	104.90 ± 4.8
3	1164 ± 62	1214 ± 20	95.87 ± 4.9
4	862 ± 56	921 ± 16	93.48 ± 5.6

## Data Availability

Not applicable.

## Supplementary Materials

The following supporting information can be downloaded at: https://www.mdpi.com/article/10.3390/bios12050331/s1, Figure S1: Scanning cycles of deposition of AuNPs; Figure S2: Comparison of the relative resistances of Ca^2+^ at different concentrations with and without NGR modification.
