# Supplementary material for: Electrochemical Biosensor Using Nitrogen-Doped Graphene/Au Nanoparticles/DNAzyme for Ca2+ Determination
Source: Biosensors (Basel). 2022 May 12;12(5):331. doi: 10.3390/bios12050331 (PMC9138538; doi:10.3390/bios12050331)
Supplement: Supplementary file 1 [file biosensors-12-00331-s001.zip › biosensors-1682670-supplementary.pdf]

Electrochemical Biosensor using Nitrogen-doped graphene/Au nanoparticles/DNAzyme for Ca^{2+} Determination

Zhixue Yu [1], Hui Wang [1], Yiguang Zhao [1], Fan Zhang [1,2] , Xiangfang Tang [1] and Benhai Xiong [1,*]

1 State Key Laboratory of Animal Nutrition, Institute of Animal Sciences, Chinese Academy of Agricultural Sciences, Beijing 100193, China; 82101205310@caas.cn (Z.Y.); wanghui10@caas.cn (H.W.); zhaoyiguang@caas.cn (Y.Z.); 82101199124@caas.cn (F.Z.); tangxiangfang@caas.cn(X.T.)

2 College of Animal Science and Technology, China Agricultural University, Beijing 100193, China

* Correspondence: xiongbenhai@caas.cn (B.X.); Tel.: +86-010-62811680(B.X.)

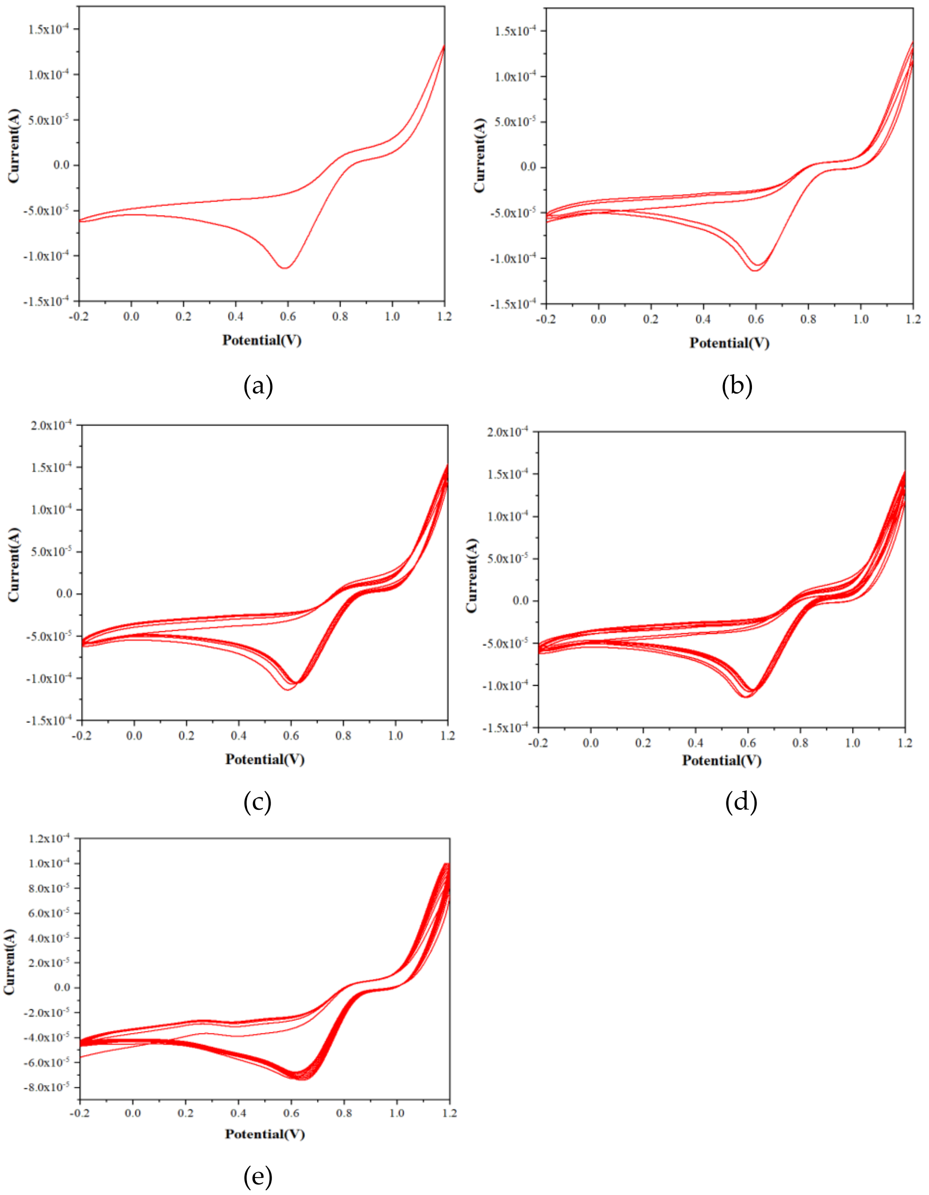

Figure S1. The images of AuNPs deposited 2 r (**a**), 5 r (**b**), 10 r (**c**), 15 r (**d**), and 20 r (**e**).

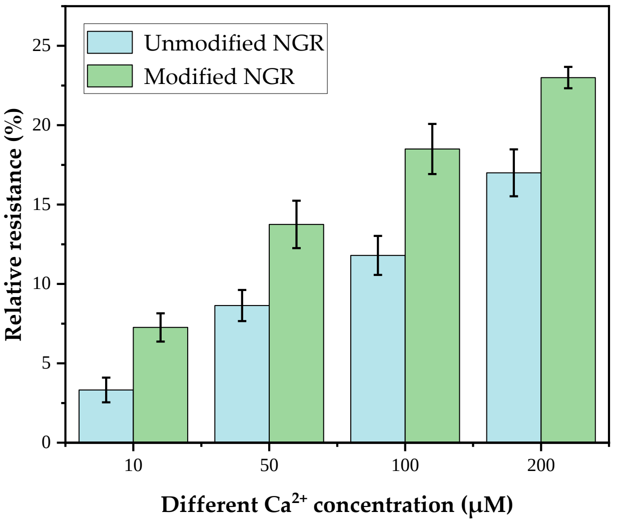

Figure S2. Comparison of the relative resistances of Ca^{2+} at different concentrations with and without NGR modification.
